# Motion Magnification Applications for the Protection of Italian Cultural Heritage Assets

**DOI:** 10.3390/s22249988

**Published:** 2022-12-18

**Authors:** Vincenzo Fioriti, Ivan Roselli, Antonino Cataldo, Sara Forliti, Alessandro Colucci, Massimiliano Baldini, Alessandro Picca

**Affiliations:** ENEA, Casaccia R. C., Via Anguillarese 301, 00123 Rome, Italy

**Keywords:** video-based technique, motion magnification, vibration monitoring, monuments

## Abstract

In recent years, the ENEA has introduced a novel methodology based on motion magnification (MM) into the Italian cultural heritage protection and monitoring field. It consists of a digital video signal processing technique able to amplify enormously the tiny movements recorded in conventional videos, while preserving the general topology of the acquired frames. Though the idea of such a methodology is not new, it has recently been provided with an efficient algorithm that makes possible a viable and low-cost magnification. Applications are extremely varied in almost every field of science and technology; however, we are interested in its application to the safeguarding of architectural heritage, a sector of the utmost importance for Italy. As ancient buildings can be extremely sensitive to even minimally invasive instrumentation, most common monitoring sensors can be replaced by contactless tools and methods, such as video-based techniques like MM. It offers many advantages: easy to use, contactless devices, virtual sensors, reusability of the videos, practicality, intuitive graphical results, quantitative analyses capability and low costs. These characteristics are well suited to the monitoring of large ancient monuments; on the other hand, historical sites have peculiarities of their own, requiring careful approaches, proper tools and trained personnel. Moreover, outdoor applications of MM present quite notable difficulties from a practical point of view, e.g., the dimensions of the studied objects, uncontrolled environmental conditions, spurious vibrations, lighting change/instability, etc. Here we give a general idea of the potential of MM and related issues, using some relevant in-the-field case studies in Italian heritage protection.

## 1. Introduction

Video-based monitoring methods offer a unique property: each pixel of a frame can be considered as a virtual sensor, which is like having a very high sensor density on the studied object surface. Another remarkable property of video-based methods is that data are acquired from a distance. This implies that hardly reachable points of non-movable objects can be analyzed as a valuable alternative to placing sensors on their surface. However, there are some movable objects that are particularly vulnerable or delicate, such as archaeological artifacts and works of art. In such circumstances, video-based methods provide a contactless solution, which guarantees the maximum respect for the preservation of the studied object. Moreover, processing techniques can be focused on specific regions-of-interest (ROIs), which means limiting the analyses to selecting the most convenient pixels instead of analyzing the whole image comprising millions of pixels. Furthermore, this allows changing the choice to a more suitable area or the selection of more useful pixels if a different analysis of the video recording is required at a subsequent time.

All the above could be applied also to the study of vibrations, which essentially are tiny motions of the studied object. A major issue for video-based analysis of vibrations is that tiny movements embedded in a conventional digital video generally require a relevant amplification of the pixel time series in order to visualize the vibration. However, such amplification should preserve the general topology of the object in the frames. After many attempts to find an effective algorithm to solve this problem, a research group at MIT finally succeeded [1], providing a viable motion magnification (MM) algorithm. It acts like a microscope for motion in video sequences affecting only some groups of pixels and unveiling motions hardly visible with the naked eye. MM uses the spatial resolution of the video-camera to extract physical properties from images to make inferences about the dynamical behavior of the object [2,3,4], and therefore it is a contactless method, offering simultaneous, quasi-continuous, measurements. If we compare the constraints of conventional vibration sensors, such as seismographs and accelerometers, with the video-based techniques [5], problems due to the need for contact, sparse discrete points, sensitivity to temperature, aggressive chemical products, measurement of absolute values, and direct acquisition arise. On the other hand, videos are affected by moving objects, sensitivity to alternating-current lighting (if indoors), reflections, fog, smoke, clouds, wind (outdoors), measurements relative to the camera value, 2D-only monitoring in case of a single camera, quantitative errors in calculations, camera displacements, and by the presence of large shifting of the object.

Nevertheless, researchers are very interested in assessing the method’s feasibility, since conventional devices are surely more precise but are also expensive and much less practical. Therefore, MM can be useful in a large number of scientific fields, and nowadays there are even hardware tools available for performing the MM elaborations almost in real-time, although the cost of these devices is quite high. Today, the effectiveness of visual methods in the industrial environment, physics, biology and engineering is established. A number of experiments conducted on simple geometries like rods and other small objects have demonstrated the reliability of this methodology inside a laboratory, compared to contact accelerometers and laser vibrometers [6,7]. Of course, MM has been used also in outdoor environments to study large objects like bridges or buildings. The most recent review of such applications can be found in [5]. However, a review of specific applications for cultural heritage assets is still not available. On the one hand, this kind of application is of utmost importance in a country rich with historic and cultural assets like Italy. On the other hand, cultural heritage assets are characterized by specific issues and challenges for investigation and monitoring systems, mainly related to the high level of protection cautions and the specific mechanical behavior of ancient materials. In fact, the Italian authorities are deeply concerned about the protection of the Italian heritage, one of the largest in the world. The huge number of sites and artifacts to be monitored and protected is a challenging task. In addition, Italy is notably affected by natural hazards, such as flooding, earthquakes and climate change. Therefore, the development of new methods to face the challenge of easily and properly monitoring such a massive amount of heritage assets is of great interest in Italy. In addition, there is the need to take into account the cost of the monitoring tools and their ease of use, given the need to provide technicians with practical and inexpensive equipment. Hence, the MM methodology appears to be a good solution for all these issues.

In the following, a review of recent studies conducted on cultural heritage assets in Italy using the MM method are presented and discussed. They were carried out to monitor and analyze structures and objects both qualitatively and quantitatively. This review also provides the elements to illustrate the potential of this innovative methodology. Such studies were carried out in the framework of approved research projects or on request by the site manager in order to explore the potential of this innovative method. The presented case studies were not selected on the basis of specific criteria other than the assumption that object vibration can be caught and analyzed by video recordings if the camera resolution, pixel dynamic range and speed used are sufficiently high in relation to the intensity and stability of lighting conditions. In addition, the challenge was to explore the possibility of providing valuable indications even with common commercial low-cost cameras.

First, some laboratory experiments using shaking table tests to validate the methodology are presented. Then, several in-the-field case studies related to some important Italian monuments will be described. In particular, studies on the Ponte delle Torri of Spoleto and several monuments in Rome (the so-called Temple of Minerva Medica, the archaeological site of the Crypta Balbi, the Colosseum, the Villa dei Quintili and the Sarcophagus of the Spouses) are illustrated.

## 2. MM Algorithms Used

In the case studies presented in this review, the Eulerian and the phase-based versions of the MM algorithm were implemented to process the videos. Subsequently, the magnified videos were subjected to further processing steps to analyze the frequency content of the signals or to extract a normalized relative displacement map of the studied objects. In fact, the main aim was to provide a rough estimate of the modal frequencies and of the relative displacements related to the deformed configuration (modal shapes), which are widely used to monitor the structural health of cultural heritage structures [8]. However, the possible applications are not limited to the estimate of modal parameters.

As for the Eulerian version of the MM algorithm, the main equation for the direction x (in a video with frame coordinates x and y) is the following [2]:

∆I = f(x – (1 + α) δ (t))(1)

where I is the pixel intensity, α is the amplification factor and δ (t) is the displacement at time t. It is worth noting that (1) is achieved by a band-pass derivation, so that the main input parameters to be tuned in the algorithm are the cut-off frequencies of the band-pass derivation and the amplification factor α. Consequently, it can be concluded that magnifying the motion displacement substantially means adding α∆(x, t) to I(x, t), assuming the validity of the Taylor’s expansion of the magnified pixel intensity Im(x, t) defined by the following:

Im(x, t) ≈ I(x, 0) + α ∆(x, t) ≈ f (x) – δ(t) (∂f/∂x) – α δ(t) (∂f/∂x) + O(ε, δ)(2)

where O is the remainder and ε is the error due to the Taylor’s approximation. As the validity of the above Taylor’s approximation is verified as long as O is small, in practice, the algorithm works efficiently for small motions in the video and small amplification α. This substantially means that a compromise must be found on the value of α to avoid excessive image distortions in the magnified video. On the other hand, vibrations are generally small oscillations in the object’s shape, so that MM is potentially very applicable to the analysis of vibrations.

The phase-based version of the MM algorithm is slightly different. In comparison to the previous Eulerian method, the phase-based version permits the use of larger α factors and is significantly less sensitive to noise [3]. More recently, a novel method using multifrequency absolute phase retrieval and fast cosine transform with extension to phase correction operation was proposed by [9]. Further advances based on Gaussian Mixture Model (GMM) learning algorithm permit it to improve the MM results with fewer artifacts and better anti-noise performance [10].

In terms of identification of the modal frequencies, of course, the Shannon–Nyquist theorem must be taken into account, according to which only frequencies smaller than the half value of the sampling frequency, i.e., the frame rate (sometimes also called camera speed) in the video acquisition, can be detected. Nonetheless, it is strongly suggested to adopt a wide oversampling of data, in the order of many times (e.g., at least five times or more) the maximum frequency of interest. This is certainly true also in the case of the MM method. To be more explicit, considering a common low-cost camera with typical values of frame rate around 24–30 fps, a rough estimate of the frequencies can be realistically achieved below 5–6 Hz and particularly interesting results can be obtained below 2–3 Hz. This means that a commercial low-cost camera is potentially capable of identifying the first modal frequency of a large building or monument, whose main frequencies are typically below 2–3 Hz, but unsuitable for smaller objects, such as museum exhibits that typically vibrate at higher frequencies. In these cases, the use of a high-speed camera can be indispensable.

In summary, the main parameters to be set are the frequency range and the amplification factor. They are specified in the details found in a short manual enclosed with the MIT software [3]. However, a precise quantification for their tuning is lacking, as the MIT researchers themselves warn. Therefore, it is advisable to collect as much information as possible on the studied object, especially about the frequency range of interest. After that, heuristic procedures may be used through different amplification values, discarding the most noisy runs. Values of the amplification factor typically range between ten and a few hundreds. As already said, too high levels of amplification introduce disturbing distortions in the magnified video. In most cases, reducing the amplification usually is enough to fix the distortions, at least partially. Thus far, the fine-tuning of the algorithm parameters is still under study. As a matter of fact, parameters are currently tuned based on authors’ experience. However, the current commercial version of the freely available MIT magnification algorithm provides for the automatic adjustment of the noise and also the tuning of the main parameters.

## 3. Laboratory Case Studies on Historic Structure Mockups

**Figure 1:**
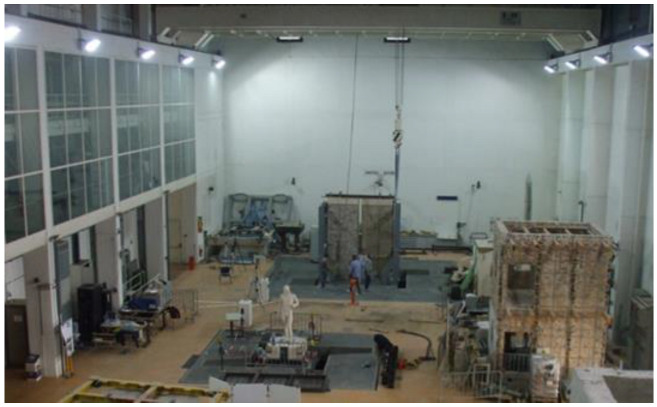
View of the ENEA Casaccia shaking tables hall. The hall lighting system by high-power floodlights is visible on top of lateral walls.

In this section, a collection of the most relevant laboratory cases carried out in Italy for the validation of MM application to cultural heritage assets is illustrated. In particular, the laboratory tests here illustrated were performed at the seismic hall facility at the ENEA Casaccia Research Centre (Figure 1), which is equipped with two 6-Degrees-of-Freedom (6-DOF) shaking tables capable of reproducing natural or synthetic seismic ground motions for structural prototypes and mock-ups up to 30 t of dead load. The largest table is a 4 m × 4 m table, with a maximum acceleration of 3 g, maximum displacement 0.125 m and a frequency range of 0–50 Hz. The laboratory is equipped with a high-resolution passive 3D optical motion capture system, called 3DVision, to track the dynamic displacements of the tested mock-ups by detecting the position of passive reflecting markers positioned at the desired measurement points [11,12]. The 3DVision system and accelerometers were used to validate the magnification methodology in indoor controlled conditions before in-the-field application to real structures in outdoor conditions.

**Figure 2:**
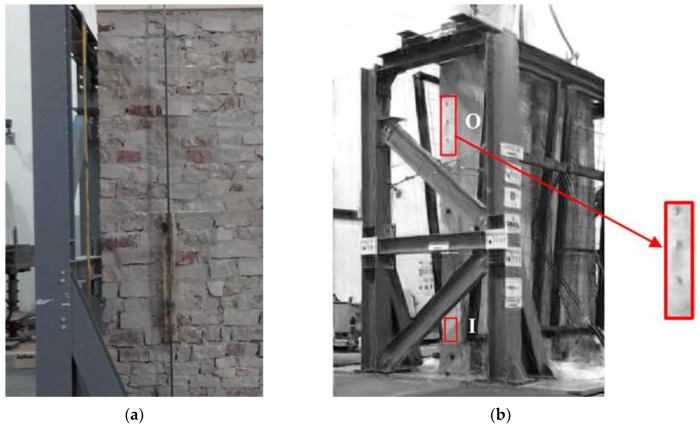
A historic masonry wall typical of central Italy tested on shaking table: (**a**) Detail of the wall during setup preparation. (**b**) The red boxes indicate the O and I ROIs used as output and input signals in the FRF, respectively. The O ROI is enlarged on the right to enhance the three markers used as reference.

Encouraging results were obtained in a shaking table test experiment for a typical Italian historic masonry stone wall [13]. In this case, a commercial tablet camera (pixel resolution 720 × 1280 and frame rate 28 fps) recorded the videos. In Figure 2, the test setup is shown, and the wall is enclosed by safety steel nets to prevent risks related to possible collapse of the stones. Of course, as it would be too cumbersome to analyze all frame pixels, and not all of them generate useful information anyway, a small convenient ROI was identified where, according to the theoretical dynamic behavior of the wall, the highest displacements were expected; preferably this is the area having the highest image light contrast, thus providing the highest signal-to-noise ratio (SNR). This ROI (indicated with O in Figure 2) was used to extract the output signal to be processed by Frequency Response Function (FRF) in comparison with an input signal, also extracted by MM in a second ROI at the base of the wall (indicated with I in Figure 2). As reference, three markers of the 3DVision motion capture system located in the O ROI were used. The 3DVision system consists of ten infrared cameras positioned around the shaking table and is able to track the displacements of each marker with accuracy in the order of 0.01 mm; therefore, velocity and acceleration can be derived by numerical methods.

Signals from the markers provided the reference benchmarks for the comparison with the MM. It is also to be noted that usually the presence of edges or texture is helpful for the MM. Unfortunately, the ROIs in this case are rather homogeneous, which causes a quite high signal noise. Signals from the magnified motion technique do not provide directly the displacements, although they could be recovered by means of the constant contours method. On the other hand, they may be used to calculate the Power Spectral Density (PSD) or the Fast Fourier Transform (FFT), allowing the modal analysis and the calculation of the FRF. Hence, the 3DVision system is a convenient reference to evaluate the MM performance in the frequency domain. The FRF was calculated using both the 3DVision markers and the MM signals from the selected ROIs.

**Figure 3:**
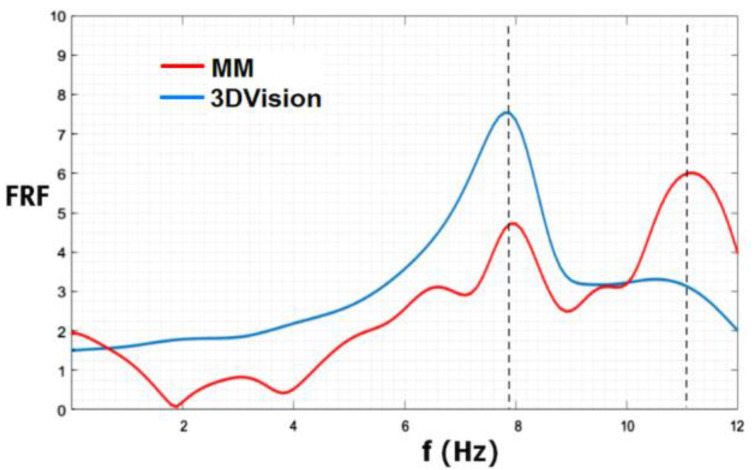
FRF magnitude by the MM technique (red line) and by 3DVision markers data (blue line).

In Figure 3, a comparison is shown between the identification of the first modal frequency by FRF frequency analysis using MM and that using the 3DVision benchmark. The MM first resonance peak was at 7.9 Hz while the 3DVision first resonance peak was at 7.8 Hz (up to a frequency resolution of 0.1 Hz). The second peak at 11.1 Hz is not reliable as the MM videos were acquired at 28 fps. For further validation, the FRF was also calculated by standard accelerometers, which provided a value of 7.89 Hz for the first modal frequency. Considering as reference the most surely reliable measurements, i.e., the accelerometer value, the 3DVision system yielded an error of −0.13% and the MM an error of −1.14%.

More recently, a series of similar shaking table tests was performed on historic masonry walls in unreinforced and reinforced conditions with several retrofitting techniques [14]. The acquisition system consisted of a commercial digital camera with 24.3-megapixel resolution, placed on a tripod at approximately 4 m distance from the specimen, recording at a rate of 30 fps. In the 17 tests carried out, the resulting frequency range was 3–7 Hz and was obtained with errors between 0.5% and 6.3%, with an average value of 1.7% compared to conventional sensors, which is in substantial accordance with the previous test carried out with similar frame rate.

**Figure 4:**
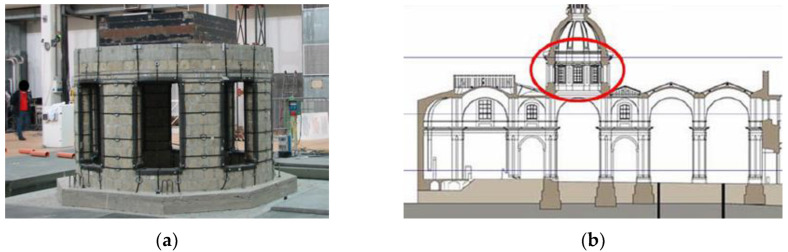
Church of San Nicolò l’Arena, Catania, Italy: (**a**) setup of shaking table test with strengthening intervention on 1:5-scaled mockup of the drum; (**b**) position of the drum (red ellipse) in the structure.

More complex structural portions of historic buildings were also tested. The mock-up of Figure 4a represents the drum of the San Nicolò l’Arena dome (scaled 1:5) subjected to a shaking table test to verify the seismic performance of a strengthening intervention [15]. The church is part of an enormous 17th-century Benedictine monastery located in Catania, Sicily (Figure 4b). This mock-up represents a quite typical architectural element that can be found in many ancient churches in Italy.

Before and after each earthquake motion test, the mock-up is excited with extremely low-intensity pseudo-white-noise vibration (random test) for dynamic identification of the structure. In random tests, the MM elaboration may enhance the deformations of the tested mock-up before much stronger vibration tests cause damage to the structure.

The video resolution was only 640 × 480. Nonetheless, the magnified video results were extremely effective in visualizing the dynamic behavior of the drum, especially in terms of modal shapes and frequencies identification. This was achieved by taking videos at 60 fps, which confirms the importance of video speed, allowing large oversampling with respect to common 20–30 fps cameras. In particular, the MM analysis was limited to the first modes (below 10 Hz), which usually are the most important in terms of mass participation, providing indications of the global behavior of the overall structure.

**Figure 5:**
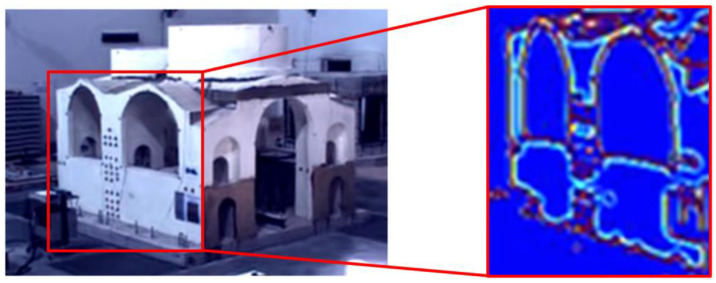
Skeletonization from the magnified video of a 1:10-scaled mock-up of Hagia Irene, Istanbul, Turkey.

Figure 5 shows a mock-up of an ancient church built under Emperor Justinian I in 548 AD. More specifically, it is a 1:10-scaled prototype of the central part of Hagia Irene [16], a church located in the outer courtyard of Topkapi Palace in Istanbul. This is not an Italian site, but the laboratory experiment was conducted in Italy and is representative of video processing potentialities applicable to Italian churches. Once magnified, the video was also processed by a computer vision technique able to obtain an optimal skeletonization of the structure, which was effective in reducing signal noise and in enhancing the most sensitive parts of the church. In particular, the skeletonization was able to emphasize geometrical and topological properties of the shape of the object. These elaborations were useful to enhance some details that are not clearly visible in the original video. This was probably due to the fact that the laboratory illumination was not specifically studied for the MM acquisitions, which requires high-power lighting for optimal MM results. In fact, at the time of this test acquisition, the laboratory was equipped with old metal-halide lamps, then replaced with more powerful LED lamps. In addition, alternating-current lighting might induce disturbances and shadows that affect the recorded signals. Furthermore, the utilized video equipment comprised quite low-resolution and low-cost cameras. Nevertheless, these laboratory tests provided the opportunity to validate the method in a controlled environment.

## 4. The So-Called Temple of Minerva Medica in Rome

**Figure 6:**
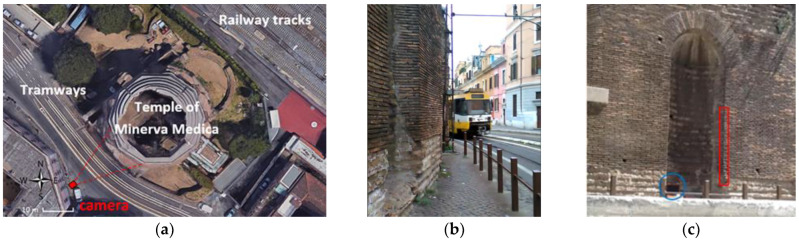
The so-called Temple of Minerva Medica: (**a**) aerial view with camera position (from Google Maps); (**b**) a tram passing by very close to the south wall of the monument; (**c**) the ROI used for extracting the MM signal is enhanced by the red rectangle at the niche corner’s edge.

The so-called Temple of Minerva Medica is located between the railway tracks and a tramway in the center of Rome (see Figure 6a), near Termini Station. Strong vibration induced by tram passages very close to the monument is clearly a major problem (see Figure 6b). The main structure was built in the late Roman Empire (early 4th century AD) as a majestic building. The main hall of the complex was probably a nymphaeum, with a large decagonal polylobate hall of 25 m width and an overall height of 32 m.

After several partial collapses and restoration interventions in the past, the size of the main hall is today almost unchanged, except for the height, which is currently slightly reduced. During the period 2016–2018, extensive monitoring campaigns were performed, combining vibration monitoring, 3D geometric laser scanning, thermal infrared and microclimatic acquisitions [14,17,18].

In this application the basic idea of the MM quantitative analysis was to take advantage of the large number of pixels contained in a frame. Theoretically, a large number of “virtual sensors”, corresponding to the number of pixels within the acquired frame, can provide a time history of intensity variation, color or grey scale. These time series contain information about the displacements of the physical points related to the pixels. Of course, not every surface of the structure generates useful information. In addition, processing all pixels of the studied object might result in a huge number of pixels requiring excessive computational time. Therefore, it is more convenient to choose a smaller ROI having the highest SNR. The identification of an efficient ROI is a crucial point in the MM procedure. However, in this case study, the authors did not use the SNR criterion; rather, an ROI containing the corner of a niche providing maximum color edge was chosen for the MM processing (red rectangle in Figure 6c). A careful selection of the pixels in the ROI can provide the MM algorithm with better signals, improving the SNR ratio and ultimately improving the final results quality. Shadows due to the angle of the sun helped the choice of a well contrasted ROI. On the other hand, very sunny exposure produces shadowed areas, which is not an optimal video illumination condition for the MM algorithm.

The video equipment utilized for this case study was a commercial smartphone with a camera at 360 × 445-pixel resolution and speed of 50 fps, which can be considered very-low-cost video instrumentation. The smartphone was installed on a tripod placed on the opposite side of via Giolitti, the adjacent street with tramways (see Figure 6a). This was the most suitable location for optimal video acquisition geometry.

**Figure 7:**
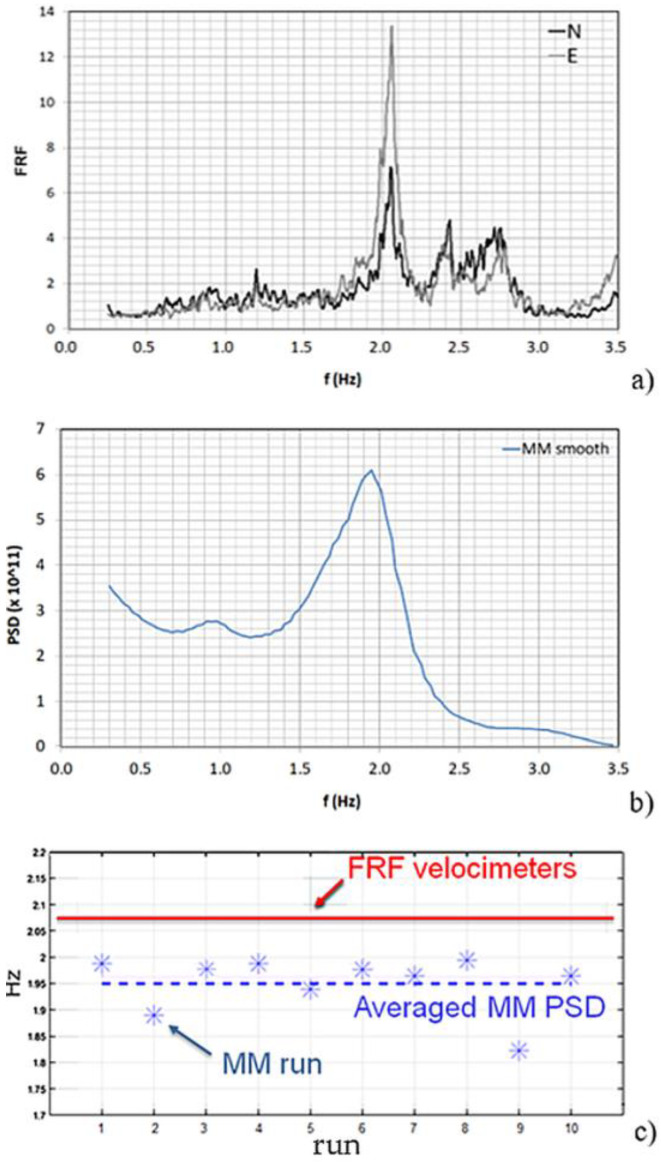
Comparison between MM and seismographs results: (**a**) the fundamental frequency identification by FRF of seismographs; (**b**) smoothed PSD of MM signal; (**c**) MM frequencies by each 10 run and average.

In addition, five seismographs were positioned on the building to estimate its FRF in order to extract the fundamental frequency of the monument (Figure 7a), which was taken as reference for the MM analysis. The seismographs were SL06 recorders (SARA Instruments co.) equipped with triaxial electro-dynamic velocimeters, set to 200 Hz sampling frequency and synchronized with GPS antenna.

Then, the PSD of the signal extracted from the MM processed video was calculated. To improve the PSD readability, a smoothing method or low-pass filtering was applied to the MM PSD, resulting in the plot of Figure 7b. It provided a fundamental frequency estimate (1.95 Hz) by averaging ten MM runs (Figure 7c), which was quite close to the one obtained from the FRF of the seismograph signals (2.05 Hz). The Welch method for the PSD calculation has been used according to indications from the MM literature [19,20,21].

This case study proved that, despite the numerous noise sources in an outdoor environment [14], and even using quite low-cost video instrumentation, MM analysis was able to identify the fundamental frequency of a historic masonry structure with interesting accuracy when the input (here provided by the tram passages) was strong enough to produce a reasonably high SNR ratio. Secondarily, the solar beams should as perpendicular as possible to the studied surface in order to provide sufficient illumination during the video shooting. Other physical limitations such as those regarding shadows, unwanted displacements of the camera, poor pixel resolution, low frame rate, a large amount of motion, distance from the object, etc., can decrease the quality of MM and must be taken into account.

## 5. The Ponte delle Torri in Spoleto

**Figure 8:**
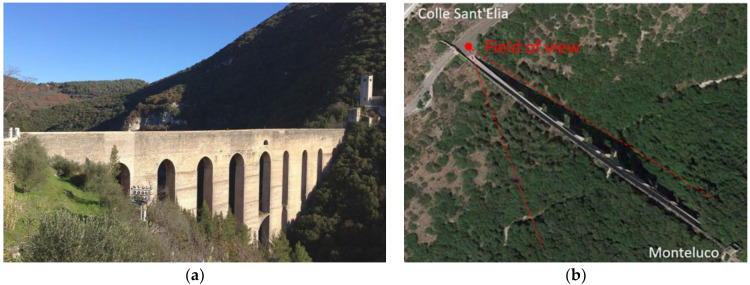
The Ponte delle Torri, Spoleto, Italy: (**a**) view from west; (**b**) aerial view of the bridge with the video acquisition geometry, with camera position and field of view enhanced in red (from Google Maps).

The Ponte delle Torri is a large historical construction that connects Colle Sant’Elia with Mount Monteluco in Spoleto, Italy (Figure 8a). It was probably built in the 13th century, possibly on Etruscan or Roman ruins. The bridge superstructure is made up of a pedestrian deck, provided with a water canal on one side, supported by lancet arcades and stone piers known as “towers”. The bridge has a state of damage typical of historic masonry bridges. At the top of the arches, especially those on the slope of Monteluco, there are heavy water infiltrations, resulting in losses of mortar binder, and wall apparatus skiving [22,23]. The main source of vibration excitation to the bridge is the wind, as no heavy traffic is present nearby.

The video equipment utilized for this case study was the same as in the experiment at the Temple of Minerva Medica. The camera was positioned on the adjacent road on Colle Sant’Elia, as shown in Figure 8b.

**Figure 9:**
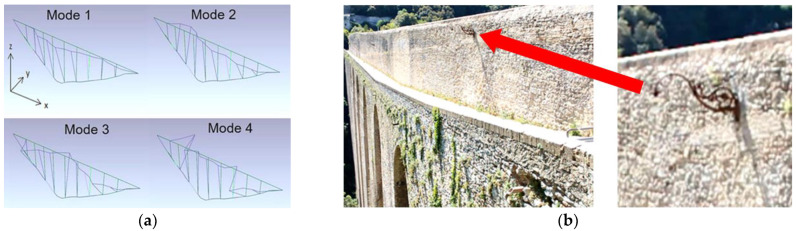
The Ponte delle Torri, Spoleto, Italy: (**a**) modal shapes of the first four modes by OMA techniques; (**b**) original frame of the recorded video with a detail of the lamppost (red arrow) on the upper wall of the pedestrian deck.

The acquisition geometry was studied for a proper detection of the first four modes of the bridge, which are transversal modes (see Figure 9a). The presence of marked edges or texture was very helpful, even if most of the wall surface is of a rather homogeneous color. As already explained, this produces a large amount of noise, which added to the environmental disturbances. Consequently, a first attempt to select an efficient ROI was to use a lamppost (Figure 9b), built into the wall about 40 m from the camera, and a portion of the pedestrian deck, which also is characterized by some well contrasted lines. The proper choice of an ROI allowed a supply of low-noise information, and it can be assumed that the lamppost vibrates with the bridge wall according to the main modes of the structure. In fact, the upper part of the lamppost spreads spurious frequencies, due to its own resonances, but it can be assumed that the lamppost local modes are at much higher frequencies than the main fundamental modes of the overall structure. However, in order to avoid the acquisition of the lamppost local modes, a further reduced ROI was successively delimited on the lamppost base attachment to the wall, where its own vibration was assumed to be more limited. Moreover, other attempts with different choices of the ROI did not seem to improve the measurement quality. Signals provided by MM contain displacement information, but they cannot be used immediately as real displacements. However, acceleration may be calculated and related PSD can be obtained. Every pixel can potentially provide a signal; therefore, an averaged PSD can be calculated over all the ROI pixels’ acceleration time series, reducing the frequency noise. A dense presence of peaks suggests a high level of noise and of course may be misleading. Therefore, only acceptably smooth PSD spectra with recognizable frequencies should be considered. In practice, to avoid a biased choice, manual selection of the ROI can be repeated several times to provide input for improved calculation run precision. In this study, ten runs of the MM-PSD procedure were adopted as a good trade-off between speed and precision (intended as closeness to the mean value).

To provide benchmark data, three SL06 seismographs acquired the ambient vibration on site [22]. Multiple Test Setups Measurement Procedures (MTSP) were performed with one common measurement point on top of the central pier of the bridge as reference sensor. As the reference sensor was in the same position during all setups, it basically measured the mode shapes in this position over and over, while other sensors were moved to different positions on the bridge. Such a reference position was determined as the measurement point where the modes of interest were supposed to have the highest response level according to FEM modal shapes. Data were acquired in eight configurations, each representing a test setup in the MTSP. All configurations were acquired for at least 20 min. OMA techniques were applied to the velocimeter data to extract the modal parameters of the bridge.

**Figure 10:**
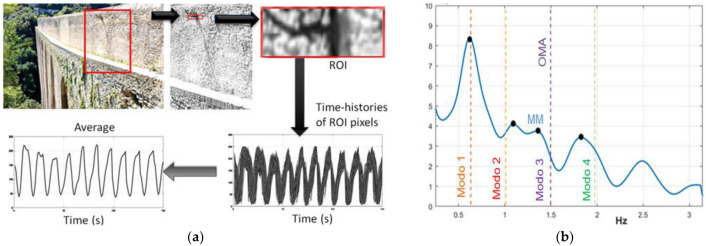
Analysis of the MM data from the Ponte delle Torri: (**a**) selected region-of-interest (ROI) and related processing flow; (**b**) typical PSD of an MM run with the first four peaks (bold black dots) compared to the modal frequencies identified through OMA of seismograph data (colored dashed vertical lines).

In this case study, more details on the practical usage of the MM algorithm [23] will be explained. To run the MIT MM algorithm, some parameters are required. In particular, the magnification frequency band and the amplification factor are the most important. A frequency range of 0.5–2.5 Hz and an amplification factor of 140 were considered. The authors were supported by previous analyses [22] in choosing the MM frequency range properly. Otherwise, one can opt to try to set a suitable band on the basis of experience. After the phase-based magnification video elaboration [3], the ROI selection was performed, meaning that only some pixels of the image were selected for further processing steps. Initially, an ROI as in the red box of Figure 10a was chosen for its morphological difference from the rest of the wall and providing relevant color edges to the algorithm.

**Figure 11:**
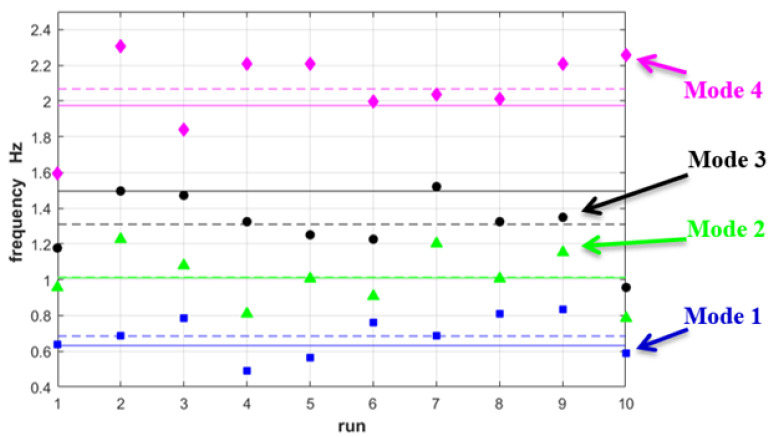
Values for the first four modal frequencies obtained by MM-PSD with 10 runs (colored dots) and average values (dashed lines) compared to OMA values (continuous lines).

Figure 10b depicts the identification of the first four modal frequencies by a typical run of MM-PSD compared to the OMA results. The smooth appearance of the MM-PSD depends also on the short time span of the video that decreases the PSD frequency resolution. Dispersion of run results is evident: statistical methods might help improve the accuracy of the average estimates. The modal frequencies calculated by MM analysis over ten runs are shown in Figure 11, where the average estimated frequencies can be compared to OMA values.

Among the several OMA techniques available, Frequency Domain Decomposition (FDD), Enhanced Frequency Domain Decomposition (EFDD) and Stochastic Subspace Identification (SSI) were utilized. EFDD provides also an estimation of modal damping, while SSI is a more sophisticated and automatic procedure based on time-domain approach [22]. The average value from all the above OMA analyses was taken as reference for the modal frequencies.

To understand why the MM method appears less accurate for the Ponte delle Torri, one should take into account the adverse environmental conditions, which prevented a correct recording angle for the camera, the distance from the target, the absence of suitable edges and, of course, the weak vibration sources. This last point seems to have been the decisive factor. MM is able to amplify sufficiently even the micro-displacements caused by the wind, but the signal-to-noise ratio remains low if the vibration sources are weak, and further increasing the amplification factor would produce many artifacts in the magnified video. In other words, the displacements are recovered, but amid a high noise level that seriously affects the MM-PSD elaborations. For more details on this case study, see [24].

## 6. The Archaeological Site of the Crypta Balbi

**Figure 12:**
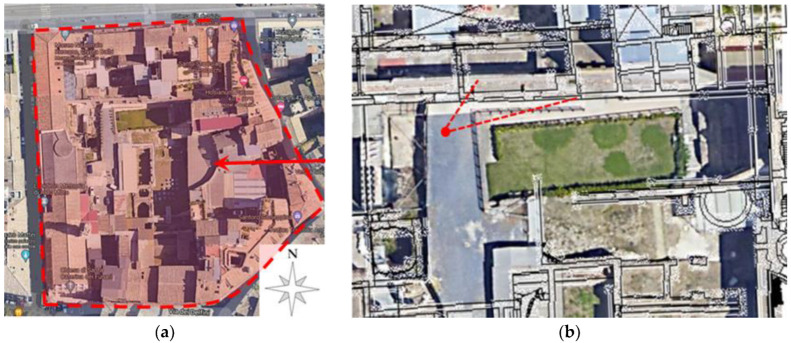
The so-called Crypta Balbi in Rome: (**a**) Aerial photo of the archeological site (from Google Maps). The red arrow indicates the exedra. (**b**) Video acquisition geometry with camera position and field of view (in red) of the 16th-century wall.

The Crypta Balbi was the theatre of Lucius Cornelius Balbus (13 BC), which included a vast hall (the so-called Crypta Balbi) and an exedra on the opposite side (Figure 12a). The maximum capacity of the theatre was of around 7000 people and had an overall diameter of about 100 m. It was quite small in comparison with the major Roman theatres of that time. However, its importance lies in the fact that it represents a unique opportunity to study how history has modified the architectural and urban environment through an impressive archaeological overlapping of remnants from ancient Rome to the nineteenth century. Over the centuries, this complex experienced the alternating of several cultural settlements. Among them were the Church of Sancta Maria Dominae Rosae, the charity hospice of St. Ignazio Loyola and other private constructions. The area of the Crypta was neglected until 1981, when the Italian government acquired the property and finally assigned it to the Museo Nazionale Romano in 1983. It is something unique in archaeology, as it gives the opportunity of visiting remnants from an incredible range of historic eras, with ruins on the same site dating from the 1stcentury BC to the 19th century.

It is well known how historical monuments in the urban environment are severely affected by anthropic vibrations, air pollution and other aggressive agents; therefore, the health monitoring of those structures is essential to preserve the sites. If the monument to be monitored is large, extended and includes many minor parts, it is not feasible to use a sensor for each studied element. On the other hand, video-based methods can provide both a global view and also a detailed local analysis when needed. In such a context, MM was tested for this purpose, obtaining interesting results.

In Figure 12b, the video acquisition geometry used for the analysis of the main 16th-century wall in the northern area of the site is shown. In particular, a recorded frame could be mapped with different colors according to the mean displacement revealed in each pixel by magnification. False color visualization could be positively utilized to indicate with immediacy the most deformed points under ambient vibration.

**Figure 13:**
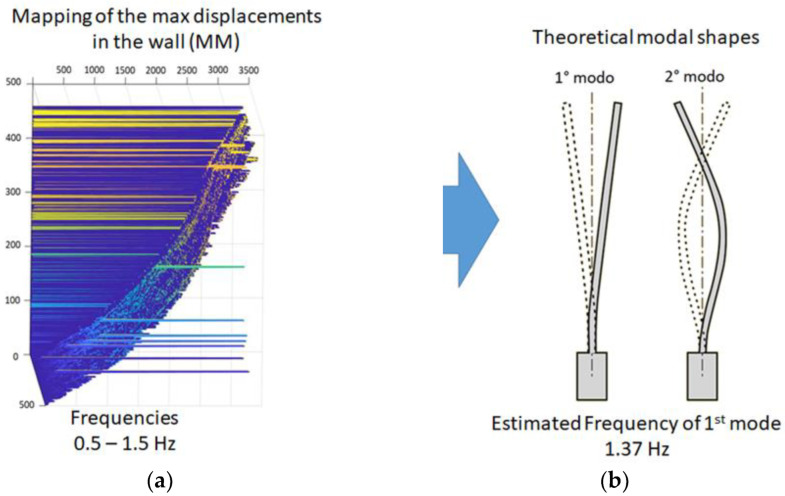
MM analysis of the 16th-century wall: (**a**) mapping of the max displacements of the wall in the frequency range 0.5–1.5 Hz; (**b**) theoretical modal shapes of a straight vertical cantilever.

The mapping of the maximum displacements of the wall magnified in the frequency range 0.5–1.5 Hz (Figure 13a) helped assess the modal shapes for the estimated first and second modes (Figure 13b).

**Figure 14:**
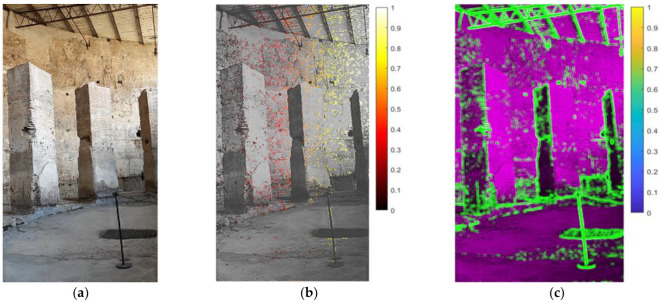
Columns of the Exedra: (**a**) original frame of recorded video; (**b**) relative normalized displacement pattern; (**c**) image entropy analysis.

Another important advantage of using video-based methods for investigating cultural heritage is the potential to examine areas of the structure that would be otherwise very difficult to reach physically without putting the operator’s safety at risk, not to mention the possibility of contactless investigation of the monuments [25,26,27]. An example of this was the analysis of the columns of the Exedra (Figure 14).

**Figure 15:**
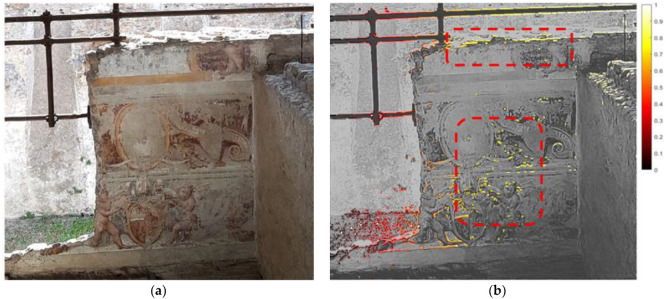
MM analysis of a fresco of the ruined church in the courtyard: (**a**) original frame of recorded video; (**b**) relative normalized displacement pattern.

**Figure 16:**
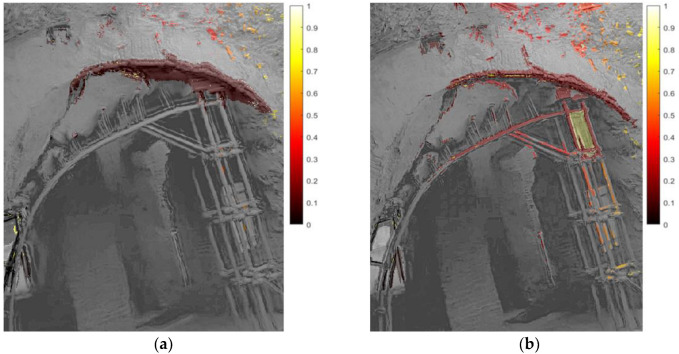
Relative normalized displacement pattern of a retrofitted arch: (**a**) with magnified frequency range 0.5–1.5 Hz; (**b**) with magnified frequency range 7–12 Hz.

The analysis of the relative normalized displacement pattern helped identify the most deformable areas, which provided interesting indications about the stability of the pattern of the frescoes’ plaster layers (Figure 15), as well as about the response of objects at different frequency ranges (Figure 16). In particular, some parts of a steel retrofitting of an arch resulted in amplified vibrations in the frequency range of 7–12 Hz.

## 7. The Colosseum

The most famous monument of Rome is located in the city center where underground subway lines, tramways and road vehicles are sources of relevant vibrations. Video shooting was carried out using a commercial camera, at a low frame rate (28 fps) and positioned at the considerable distance of about 100 m from the monument.

**Figure 17:**
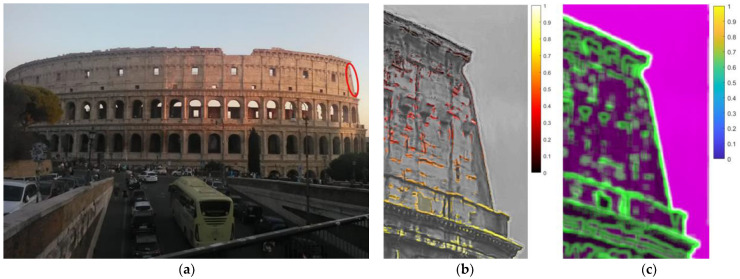
Colosseum: (**a**) Heavy road traffic around the monument. In addition, a subway station is located just beneath the trees on the right. The red area indicates the ROI considered for MM analysis. (**b**) Relative normalized displacement pattern after MM within the frequency range 1.0–1.2 Hz and α equal to 100. (**c**) Image entropy analysis.

In Figure 17a, a red ellipse indicates the analyzed ROI, which was selected in an area with a sharp edge in the image contrast so that a good signal for the magnification processing would be provided, as confirmed by the image entropy analysis shown in the following. The ROI comprised a recently consolidated part of the external wall of the Colosseum.

The video was magnified at frequencies from 1 Hz to 5 Hz, corresponding to the main modes described in the literature [28,29]. Visual inspection of the magnified videos confirmed that the ROI area provided a higher response at the lowest frequencies in the analyzed range, corresponding to the first mode at 1.03 Hz. Consequently, the MM analyses were focused on the frequency range 1.0–1.2 Hz. In particular, in Figure 17b, the mapping of the relative normalized displacement pattern after MM within the frequency range 1.0–1.2 Hz is reported.

The amplification factor was set to a quite high level (α equal to 100) because of the considerable distance from the studied object. Nonetheless, the good lighting conditions (stable and sunny conditions) made it possible to avoid excessively disturbing noise and distortions in the MM analyses. Furthermore, concentrating the MM analyses on very low frequencies in relation to the frame rate (corresponding to an oversampling of up to around 20 times the frequency of interest) helped the quality of the results.

The complexity of the pixels’ signals was analyzed in terms of image entropy, which provided, as expected, remarkably high values along the structural edges (Figure 17c), confirming that it was a favorable choice of ROI for accurate structural frequency detection. Moreover, the selected ROI showed a good relative stability compared with the surrounding areas.

## 8. The Villa of the Quintili

**Figure 18:**
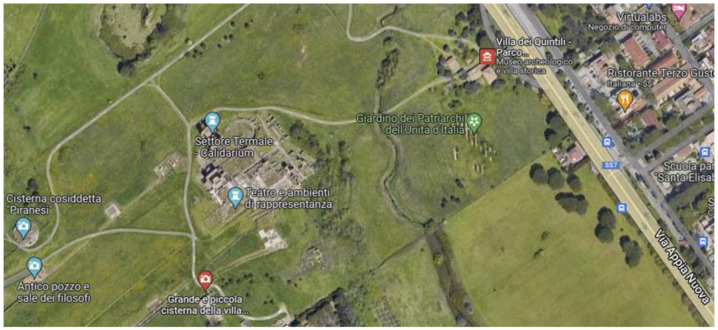
Aerial view of the Villa of the Quintili in the Appia archaeological park (from Google Maps). The *Calidarium* building can be seen in the central area. The yellow line indicates Via Appia Nuova, the closest modern highway (on the right).

Just outside the traditional boundaries of Rome and along the ancient Appian Way, the ruins of the *villa suburbana* (country house) of the Quintili represents one of the largest Roman villas ever discovered (Figure 18). The villa belonged to the Quintili brothers (around 120–180 A.D.), members of one of the richest families of the time. It was later confiscated by Emperor Commodus.

**Figure 19:**
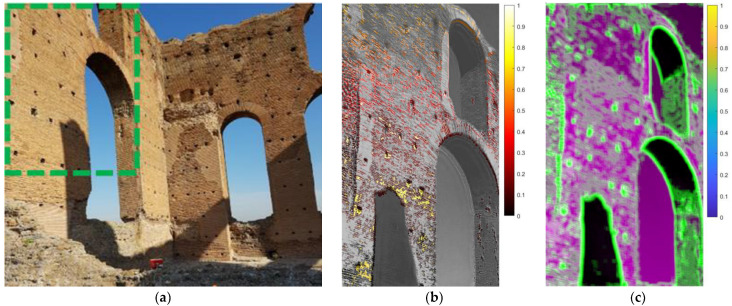
Studied wall of the *Calidarium* building at the Villa of the Quintili: (**a**) The green dotted rectangle represents the analyzed ROI. The red instrument at the base of the wall is a velocimeter for ambient vibration acquisition. (**b**) Relative normalized displacement pattern after MM within the frequency range 0.6–4.0 Hz and α equal to 35. (**c**) Image entropy analysis.

The study focused on the ruins of the *Calidarium* and *Frigidarium*, two huge buildings of the bath and the largest structures in the archaeological area. Visual inspections and ambient vibration testing using velocimeters suggested that the *Calidarium* building shown in Figure 19a was probably the most vulnerable to vibration in the entire area. The walls are about 8 m high. The main vibration sources were connected with the heavy road traffic on the via Appia Nuova, a modern highway located at a distance of about 330 m north-east from the *Calidarium* wall. Therefore, a video of the northeast wall was taken and magnified in order to detect possible induced vibrations and related wall deformations with greater precision. In particular, the green dotted rectangle in Figure 19a represents the analyzed ROI.

Videos were taken from the north-west, on a sunny day, with air temperature in the range 22–26 °C. The equipment used was a commercial tablet with a camera at 29 fps, 700 × 1000 pixels, placed at a distance of 32 m from the studied wall. Even if the surrounding vibration sources are not very concerning, the height of the studied walls are remarkable. This makes these structures quite slender and vulnerable to vibrations. Therefore, the walls were investigated with ambient vibration measurements in order to understand the dynamic behavior of the structure. In addition, the structural danger represented by earthquakes cannot be excluded, even if the seismicity of the area is only moderate.

Simple visual inspection of the MM video unveiled the parts of the wall whose deformations seemed greater than the rest of the structure. The analyzed ROI (green dotted zone in Figure 19a) was processed to extract the relative normalized displacement pattern after MM within the frequency range 0.6–4.0 Hz and α equal to 35 (Figure 19b). The frequency range was chosen in accordance with the frequencies detected through the ambient vibration testing with the velocimeters.

In this case, the amplification α was equal to 35, which is a quite modest value. Nonetheless, the MM analyses managed to provide quite clean and polished results with interesting indications about the wall behavior. This was probably possible because of the very good lighting conditions (very stable and remarkably sunny) and being able to acquire the video from a good point of view and a relatively short distance.

The obtained image entropy map (Figure 19c) enhances the most suitable pixels for frequency detection of the wall. Interestingly, the effect of the shadows of external objects on the wall that might be erroneously identified as favorable areas should be highlighted. They should be avoided because their obvious contrast lines do not follow the structural motions of the wall rigidly and thus properly.

## 9. The Sarcophagus of the Spouses

**Figure 20:**
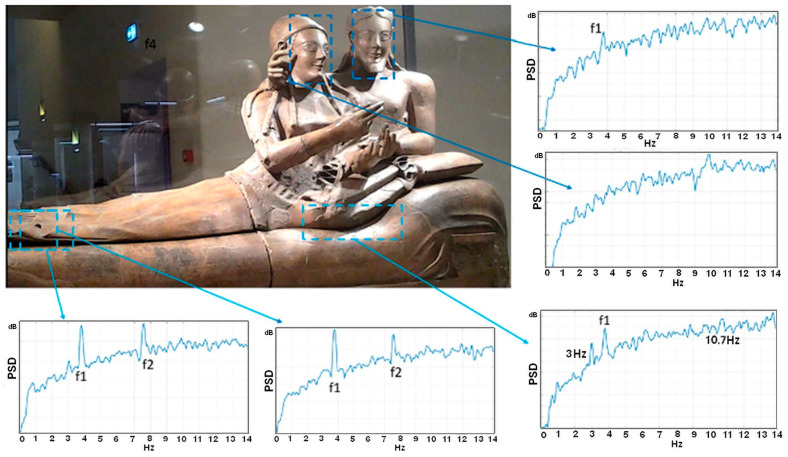
Frequency response analysis of various ROIs (blue dotted areas) on the Sarcophagus of the Spouses.

As was the case also with the so-called Minerva Medica temple, the National Etruscan Museum is located in the city center of Rome, close to tramways and trains whose vibrations represent a serious threat to the artworks preserved in the museum. In particular, one of the most important showpieces of the museum is surely the famous Sarcophagus of the Spouses (Figure 20), a masterpiece of the Etruscan art of the 6th century BC. It is made up of four parts comprising more than 400 fragments of terracotta found at the Banditaccia necropolis in Caere in 1881. Unfortunately, the Sarcophagus room is located in a position within the Museum building which is very near a busy road with tramways and just a few meters above an underground railway, both inducing strong vibrations in the room’s floor. Because of the relevance of the Sarcophagus and its potential vulnerability to vibrations, a study within the framework of the Monalisa Project funded by Regione Lazio, within the Technological District for New Technologies applied to Cultural Heritage (DTC) Program, is under way to design a proper vibration isolation system capable of protecting this artwork. The base isolation system will be designed on the basis of the dynamic characterization of the Sarcophagus. However, this could not be achieved by using conventional vibration sensors that require being physically attached to the statue, which was not allowed for such ancient works of art. Instead, video-based methods could be conveniently applied in this case, as they are contactless methods. In this context, the MM analysis was very useful for the comprehension of the dynamic behavior of the Sarcophagus.

More specifically, the statue lies on a wooden-covered support whose structure is constituted by a shallow metal bedstead-like frame. The dynamic behavior of this support was also characterized to understand its impact on the overall Sarcophagus. According to experts, the feet could be one of the most vulnerable parts. Indeed, this was confirmed by preliminary analysis which revealed two anomalous peaks in the frequency domain, as shown in Figure 20. Further detailed analyses focused on the forearms and the hands, which are possibly also extremely vulnerable parts. A notable aspect of this work was the study of the influence of the indoor lighting conditions on the final results. For this purpose, several acquisitions were made under different lighting conditions. The presence of display cabinet glass reflecting acquired light needed to be taken into account. To this end, the use of a black anti-reflection tarp in the background was experimented, as was the use of markers attached to the glass to study the display cabinet dynamic behavior. Further validation tests are currently under way with the aim of comparing the cabinet frequencies identified by MM with the ones derived from the conventional vibration sensors used as reference.

## 10. Conclusions

Some distinctive features of MM in outdoor environments and being applied to cultural heritage came up during the fieldwork carried out in recent years. In some cases, the imperative necessity to preserve a precious or vulnerable object can be a limitation even for contactless investigation technologies. A good example is the Sarcophagus of the Spouses, where the display cabinet glass lighting reflections were very disturbing for the video recording quality. The use of available low-cost and quality instrumentation, instrumentation vibration isolation, the effects of lighting conditions, etc., caused further specific issues for video recording quality, which required careful attention when designing proper monitoring systems. In some circumstances, the monument location, size and geometry can make it difficult to record videos from an optimal point of view or distance, and with proper ambient lighting conditions, when both artificial and natural light are feeble and indoor space is rather limited. The amplification factor, determining how enlarged the motions in the video are, remains crucial and thus it must be carefully selected in order to obtain good results. Unfortunately, there is still no exact procedure for a fine tuning of the amplification factor and it is currently based on operator experience. However, some simple rules of thumb may be offered. If the original video is of good quality, meaning that the video has been made with a high frame-rate camera under bright and stable lighting, and no disturbing shadows or large movements are present, then the amplification can be enlarged to quite high levels until typical distortions, similar to a disturbing haze, appear in the magnified video. On the other hand, the geometrical acquisition configuration also plays a relevant role, as is usual in video-based techniques, as well as the intensity of the vibration that excites the studied object. Briefly, if the signal-to-noise ratio is favorable, i.e., the ambient vibration induces quite ample oscillations in relation to the camera distance and angle, usually the amplification can be raised. Otherwise, particular care must be taken to prevent noise. In the case of the Colosseum, the underground subway transferred strong vibrations to the monument as well as to the camera, adding significant disturbances to the magnified videos. Thus, a complete standardization of the analysis procedures is a hard task. However, it is worth noting that, unlike most common industrial and engineering applications, vibration monitoring for cultural heritage requires less stringent constraints in standardized measurements; therefore, the trade-off between the precision/accuracy and the instrumentation’s ease of use is actually favorable to MM. At the same time, the huge number of contactless measurements available from digital video-based methods like MM finally results in an extreme flexibility not easily available with the other, more accurate techniques. Moreover, video-based technologies are very promising in terms of their development in the near future, so that the availability of more real-time and easy-to-use hardware systems, able to fix the current signal noise issues and enabling a more intensive use of MM, is likely, overcoming the most common current limitations. Consequently, the already interesting performance in the recent experiments presented here demonstrates that relevant developments in the near future have real potential to produce breakthroughs in the field.

It is also worth noting that up to now, only relatively few researchers have been dedicated to the development of the MM methodology, and in particular to the development of its application to the study of cultural heritage assets. This is true also in Italy, where the protection of culture heritage is of utmost relevance. Considering all of the above, the increasing interest in this technique will predictably see a fast acceleration in the spread of applications in this field, triggering a virtuous cycle promoting further advances and experiences.

## Data Availability

The study did not report any data.
